# An Improved DeepLab v3+ Deep Learning Network Applied to the Segmentation of Grape Leaf Black Rot Spots

**DOI:** 10.3389/fpls.2022.795410

**Published:** 2022-02-15

**Authors:** Hongbo Yuan, Jiajun Zhu, Qifan Wang, Man Cheng, Zhenjiang Cai

**Affiliations:** College of Mechanical and Electrical Engineering, Hebei Agricultural University, Baoding, China

**Keywords:** grape black rot, semantic segmentation, DeepLab V3+, channel attention, feature pyramid network

## Abstract

The common method for evaluating the extent of grape disease is to classify the disease spots according to the area. The prerequisite for this operation is to accurately segment the disease spots. This paper presents an improved DeepLab v3+ deep learning network for the segmentation of grapevine leaf black rot spots. The ResNet101 network is used as the backbone network of DeepLab v3+, and a channel attention module is inserted into the residual module. Moreover, a feature fusion branch based on a feature pyramid network is added to the DeepLab v3+ encoder, which fuses feature maps of different levels. Test set TS1 from Plant Village and test set TS2 from an orchard field were used for testing to verify the segmentation performance of the method. In the test set TS1, the improved DeepLab v3+ had 0.848, 0.881, and 0.918 on the mean intersection over union (mIOU), recall, and F1-score evaluation indicators, respectively, which was 3.0, 2.3, and 1.7% greater than the original DeepLab v3+. In the test set TS2, the improved DeepLab v3+ improved the evaluation indicators mIOU, recall, and F1-score by 3.3, 2.5, and 1.9%, respectively. The test results show that the improved DeepLab v3+ has better segmentation performance. It is more suitable for the segmentation of grape leaf black rot spots and can be used as an effective tool for grape disease grade assessment.

## Introduction

Grapes are one of the most grown economic fruits in the world. Grapes are often used in the production of wine, fermented beverages, and raisins (Kole et al., 2014). In the cultivation of grapes, the larger the area planted, the larger the scale of damage when a disease occurs as well as the greater the economic losses caused. Black rot, which is a fungal disease, is one of the most important grape diseases in the world (Molitor and Berkelmann-Loehnertz, 2011). Black rot spots are black in color and have a small spot area compared to grape leaves. Generally, the assessment of black rot damage on grapes is done by judging the size of the spot on the leaves. This operation is currently performed mainly by hand. However, the manual assessment of spot size and leaf damage area is highly subjective, difficult to quantify, and inefficient. The use of computers and image processing techniques for the identification and segmentation of black rot spots on grapevine leaves can facilitate rapid and accurate assessment of damage for targeted treatment, which is important for ensuring grapevine yield and growers’ economic incomes.

The methods of image segmentation have experienced three basic stages from classic segmentation methods, machine learning method, and deep learning method with the development of image processing and computer technology. These methods have been applied in agricultural disease detection. The classical image segmentation, such as threshold segmentation (Mehl et al., 2002; Kim et al., 2005), usually uses color and texture features (Samajpati and Degadwala, 2016) to separate the disease spots from the background. Chaudhary et al. (2012) transformed the RGB image into CIELAB, HIS, and YCbCr color space according to the different color features between the disease spots and leaf, respectively. Then the disease spots were segmented with threshold calculated by the OTSU method based on color features. Ma et al. (2017) achieved segmentation of disease spots from the background by fusion features of the super red index, the H-component of HSV, and the b-component of color space for the greenhouse vegetable images with 97% accuracy. Jothiaruna et al. (2019) proposed a method that integrated color features and region growing for the segmentation of leaves disease spots with an average segmentation accuracy of 87%. Sinha and Shekhawat (2020) segmented peacock disease spots on olive leaves according to the different textures of the leaves and spots, and the purpose of disease detection was realized. The classical image segmentation methods require high image quality, and the recognition result will be poor or even invalid if the environmental conditions changed when the image acquiring. Therefore, the generality and robustness of those methods are unsatisfactory, and the accuracy in practical application is not guaranteed.

With the development of machine learning, many researchers began to try to apply it to disease spots segmentation to improve the accuracy and robustness of segmentation. Zhou et al. (2014) inputted the color histogram of the image into the support vector machine (SVM) model to segment the Cercospora disease spots for sugar beet, and the average accuracy, recall, and *F* value were more than 0.87. Bai et al. (2017) used a fuzzy C-means algorithm for segmentation of cucumber leaves spots disease in complex backgrounds, and the experimental results showed that the average error did not exceed 0.12%. Pan et al. (2019) segmented pear blackspot disease in hyperspectral images using SVM with an overall accuracy of 97.5%. Singh (2019) applied a particle swarm optimization algorithm for the segmentation of downy mildew spots in sunflower leaves with an average accuracy of 98%. Appeltans et al. (2021) removed soil pixels from hyperspectral images by linear discriminant analysis classification and used a logistic regression supervised machine learning classifier for pixel classification of leek leaves to segment the spots of leek white tip disease with an accuracy of 96.74%. Machine learning methods can achieve satisfactory segmentation results using small sample size, but these methods require multiple steps of image preprocessing and are relatively complex to execute. In addition, the machine learning-based segmentation methods are relatively weakly adapted to unstructured environments and need researchers to manually design feature extraction and classifiers, which makes the work more difficult.

With the improvement of computer hardware performance, deep learning has been developed rapidly (Lecun et al., 2015). Common deep learning algorithms are full convolutional neural network algorithm (FCN; Long et al., 2015), DeepLab (Chen et al., 2017), U-Net (Ronneberger et al., 2015), V-Net (Milletari et al., 2016), USE-Net (Rundo et al., 2019), SegNet (Badrinarayanan et al., 2017), etc. Lin et al. (2019) designed a semantic segmentation model based on convolutional neural network (CNN) for pixel-level segmentation of cucumber leaves powdery mildew disease spots, which provided a valuable tool for cucumber breeders to assess the severity of powdery mildew. Jiang et al. (2020) combined deep learning and SVM to segment the leaves disease images of four rice species with an accuracy of 96.8%. Wang et al. (2021) used DeepLab v3+ and U-Net methods to segment disease spots from cucumber leaves, and calculate their damage levels with an average accuracy of 92.85%. Lin et al. (2019) constructed a U-Net-based semantic segmentation model for cucumber powdery mildew spots segmentation with an average accuracy of 96.08%. Wspanialy and Moussa (2020) used U-Net neural network for segmentation of tomato leaves and spots in leaves with an average accuracy of 98% and then assessed the disease hazard level. Hu et al. (2021) segmented tea leaves and disease spots using a CNN and assessed the damage level. Liang et al. (2019) used PD^2^SE-Net neural network to segment plant disease spots areas and assessed their damage levels with an overall accuracy of more than 91%. The deep learning approach has all the work done by the CNN, which does not require too much pre-processing process or artificial selection of potential features compared to classical image processing methods and machine learning methods. The deep learning approach not only reduces the difficulty of plant leaves spots segmentation but also has higher accuracy and robustness.

Our group has developed a method to improve the recognition accuracy for grape leaf black rot by combine image enhancement technology and a deep learning network (Zhu et al., 2021). It can recognize the disease spots and calculate the number, but cannot segment the disease spots from the background. To realize the spot segmentation of grape leaf black rot, this paper designs a CNN based on an improved DeepLab v3+.

## Materials and Methods

### Dataset and Test Environment Setup

The open dataset Plant Village (Hughes and Salathe, 2016) was used to perform experiments in this work, which provides symptoms of 26 common diseases on leaves of 14 plant species with a total of 54,309 RGB images. We selected 1,180 images of grape leaves infected with black rot as test subjects, and all these images were confirmed by researchers studying grape diseases. The selected images were taken in an indoor environment with a uniform gray background, and each image included only one frontal view of a grape leaf with 256 × 256 pixels. The areas of disease spots were manually labeled by LabelMe (Russell et al., 2008) software. The average number of diseases present in an image was around 15, with more than 17,000 segmentation targets present in total. Before the experimental training, 1,180 data images were divided into training and test sets, and 1,072 images were selected for training the network and 108 images were selected as the test set for evaluating the network, which was named TS1. Furthermore, to increase the credibility of the model, a large number of images of grape leaves with disease spots from orchard sites were collected *via* the Internet. A total of 108 images of grape leaves with black rot spots in natural environments were selected by researchers studying grape diseases for an extra test set, which was named TS2. During the process of network training, the training set was divided into two parts in the form of training and validation data. The division ratio of training and validation data was 9:1. The training data were used for model fitting, and the validation data were used to adjust the super parameters of the model and to preliminarily evaluate the ability of the model. The test set was used to evaluate the generalization ability of the final model. In this study, the number of epochs was 120, the input batch was four, the learning rate was 0.001, and the size of the input image was 512 × 512. The VOC 2007 dataset format was used for the dataset. The experiments were conducted on Windows 10 with the Pytorch deep learning framework. The test computer contained an 8 GB GPU GeForce GTX 1070Ti and an AMD Ryzen 51600X Six-Core processor. Python language was used for programming.

### Segmentation Method of Grape Leaf Black Rot Spots

To improve the segmentation performance of grapevine leaf black rot spots, a deep learning network based on the DeepLab v3+ was constructed. It is the third version of DeepLab, with high segmentation effectiveness and speed. In the improved DeepLab v3+ network constructed in this paper, the residual part in the backbone network ResNet101 incorporates a plug-and-play attention mechanism module. This improves the performance of various CNNs without increasing the complexity of the model. Moreover, a feature fusion branch based on a feature pyramid network (FPN) was added to the DeepLab v3+ encoder, which performs feature fusion on high-resolution and low-resolution feature maps. Finally, in the improved DeepLab v3+, one 4-fold up-sampling is replaced with two 2-fold up-sampling. Furthermore, the continuity of pixels in the obtained images is stronger and the network segmentation effect is improved.

### Channel Attention Module

**Figure 1:**
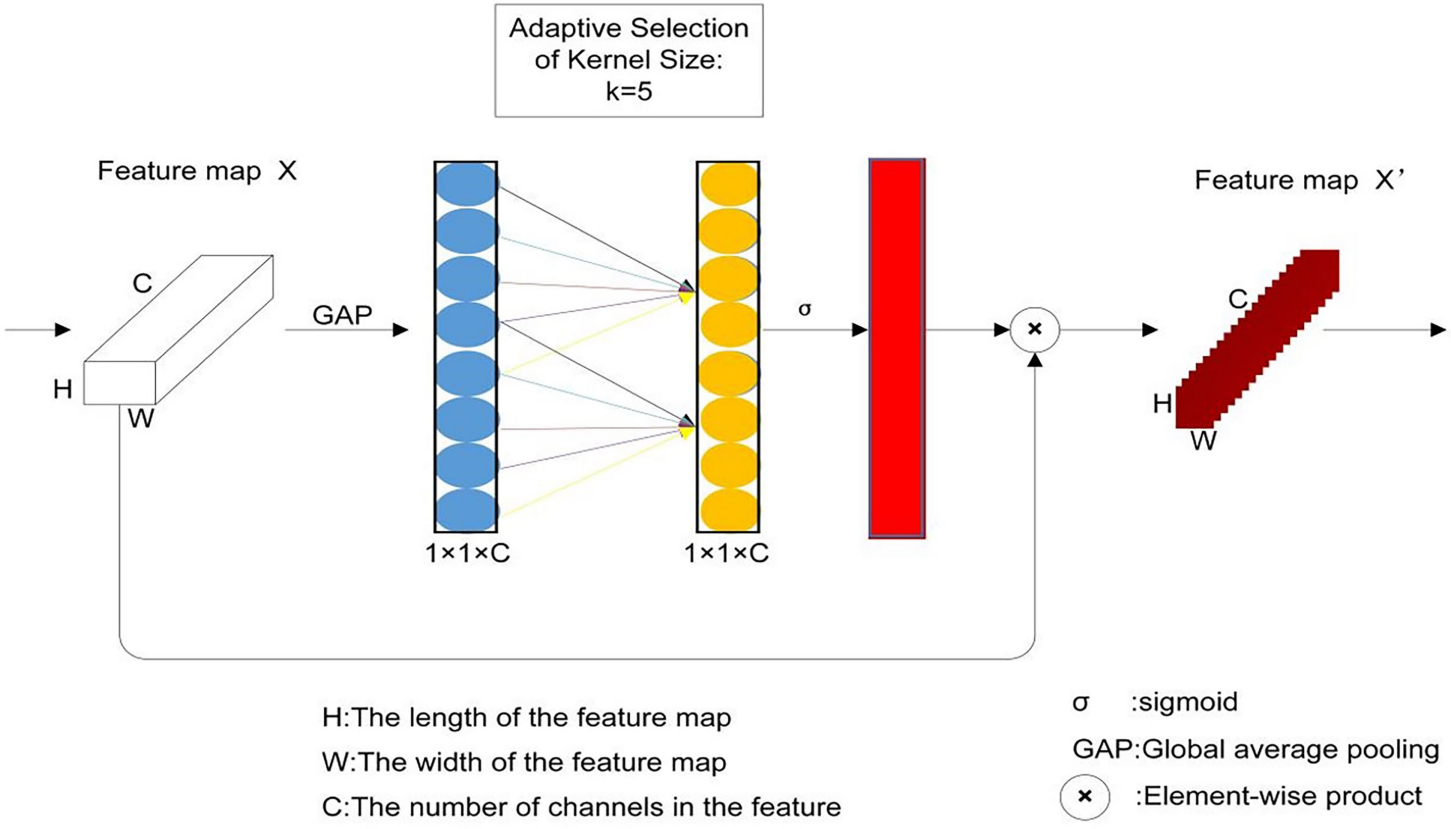
Efficient channel attention module.

The efficient channel attention (ECA; Wang et al., 2020) module is a local cross-channel interaction strategy without dimensionality reduction, which can be efficiently implemented *via* one-dimensional (1D) convolution. The ECA module is obtained by improving on Squeeze-and-Excitation (SE; Hu et al., 2020), which is an effective channel attention learning method. It predicts a weight to be weighted for each output channel. The SE method first uses global average pooling (GAP) for each feature channel individually to reduce the two-dimensional feature channel to a real number. Then, two fully-connected layers capture the non-linear cross-channel interaction. Finally, a Sigmoid function generates the channel weights with a value between 0 and 1. This weight is added to the feature channel as a weight to generate the next level of input data. The characteristic of SE is to use the correlation between channels instead of the correlation in the spatial distribution. By controlling the magnitude of the weight, the important features are enhanced and the unimportant features are weakened so that the extracted features are more directional. Compared with SE, the improvement of ECA is that the GAP operation of feature channels does not reduce the dimensionality. Instead, it captures local cross-channel interaction information by considering each channel and its K nearest neighbors. The ECA module can be used as a very lightweight plug-and-play module to improve the performance of various CNNs (Gao et al., 2020; Wang et al., 2020). Its implementation process is shown in Figure 1. The blue part uses GAP to aggregate convolutional features without performing dimensionality reduction operations. The ECA module can be efficiently implemented *via* a 1D convolution of size *k*, where the size of the convolution kernel *k* represents the coverage of local cross-channel interaction, that is, how many neighbors near the channel participate in the attention prediction of this channel. Wang et al. (2020) studied the *k* value of the CNN network with ResNet-101 as the backbone, and the *k* of the ECA module was set to 3, 5, 7, and 9 for training. The accuracy value was used to evaluate the effect of *k*. The experimental results showed that the accuracy was 78.47%, 78.58%, 78.0%, and 78.57% corresponding to the *k* value of 3, 5, 7, and 9, respectively. Therefore, *k* was set to 5 in this paper. The yellow part is the result of implementation *via* 1D convolution, and then the Sigmoid function can be used to generate the channel weights to obtain the normalized weights between 0 and 1. Finally, the original feature image X, whose matrix size is H × W × C, is multiplied by the weight generated by the Sigmoid function to obtain a new feature image X′, and the matrix size is H × W × C.

**Figure 2:**
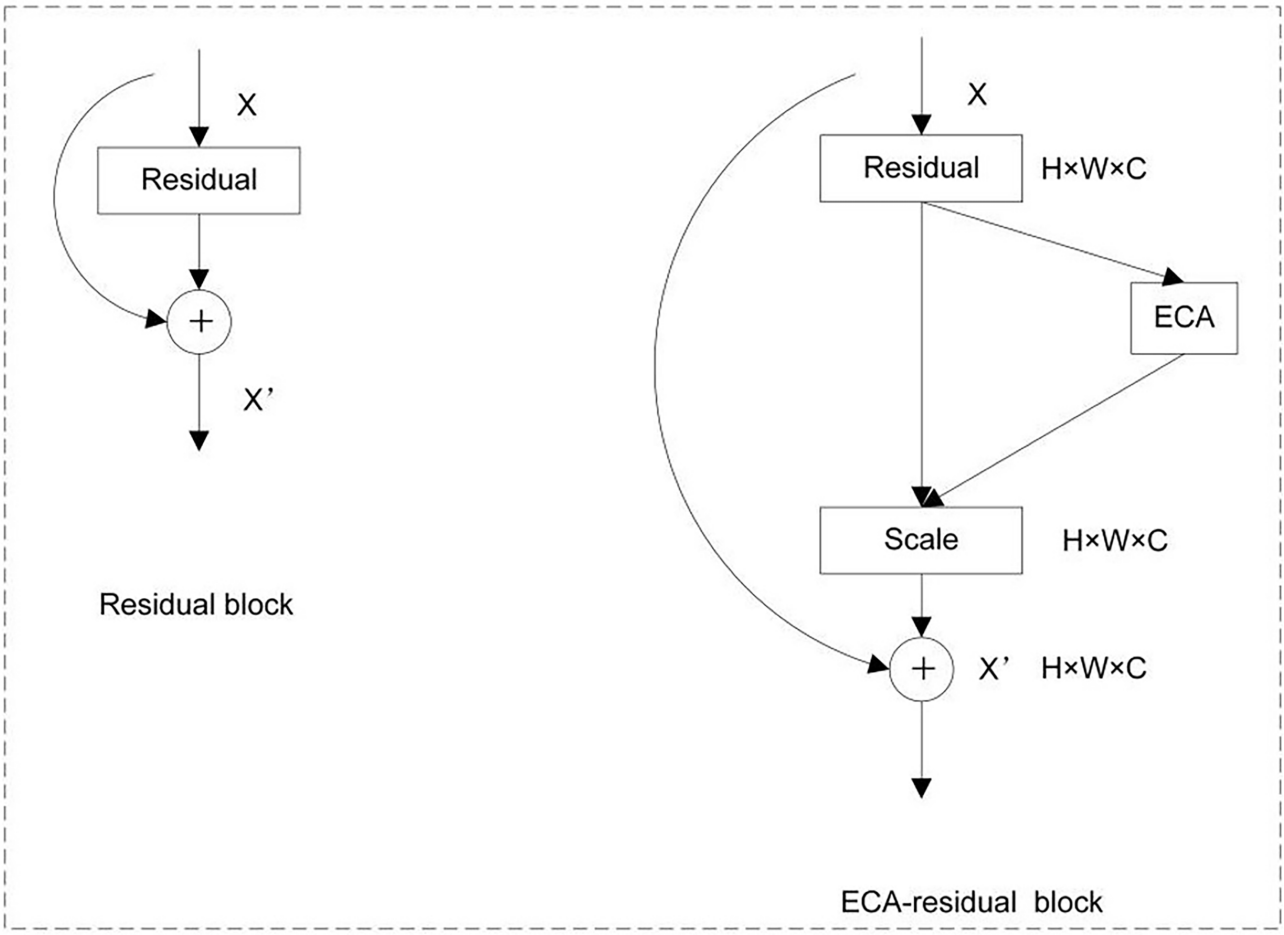
Application of the ECA module in residuals.

In this method, the backbone network of DeepLab v3+ is constructed using ResNet101, and an ECA module is inserted into the residual (Bottleneck; He et al., 2016) module of ResNet101. This method can realize the adaptive adjustment of the convolution kernel size in the channel of each residual block. The purpose is to improve the segmentation effect of the model. Figure 2 shows a schematic diagram of the insertion of ECA in the residual module of ResNet101.

### Feature Fusion Branching Based on a FPN

**Figure 3:**
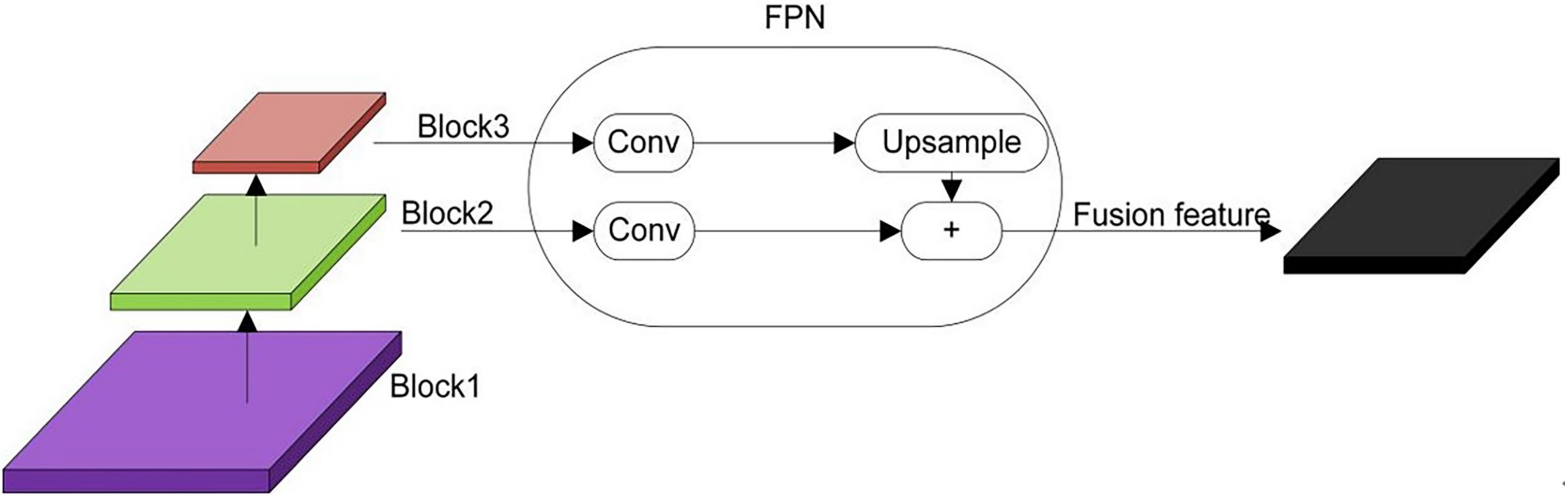
Feature pyramid execution process.

In the process of learning image features by CNNs, the resolution of the image is gradually reduced due to the deep convolution operation, resulting in low-resolution deep features at the output. In this way, there will be recognition errors for objects with a relatively small proportion of pixels in the image. The accuracy of multi-scale detection can be improved if the features at different levels of the network training process can be combined. An FPN (Lin et al., 2017) is a method that can fuse the feature maps of different layers. Feature maps that can reflect semantic information at different scales can be obtained through the fusion of FPNs. The feature fusion process of feature pyramids is shown in Figure 3. As shown, the left side is the feature maps of three different layers, whose resolutions become smaller from the bottom to the top. The middle part is the FPN, which can up-sample the deep-level features to convert them to the size of the shallow-level feature map and then fuses them with the shallow-level features. The right side is the feature map obtained after the FPN, which contains not only the deep level features but also the features of different levels. Here, the feature maps generated by Block3 and Block2 in the backbone network ResNet101 of DeepLab v3+ were fused. The feature map sizes of Block3 and Block2 were 1/16 and 1/8, and the number of channels was 1,024 and 512. In the FPN, the feature maps in Block3 and Block2 were subjected to 1 × 1 convolutional dimension reduction. The number of feature map channels in Block3 was changed from 1,024 to 256, and the number of feature map channels in Block2 was changed from 512 to 256. Then, the feature map of Block3 was up-sampled by a factor of 2 to change the size of the feature map from 1/16 to 1/8. Finally, the feature maps of Block3 and Block2 were combined to obtain the fused feature maps. The fused feature map has richer semantic and spatial information because it contains features from both levels, which can improve the segmentation effect of DeepLab v3+ network.

### Improved DeepLab v3+ Network Structure

**Figure 4:**
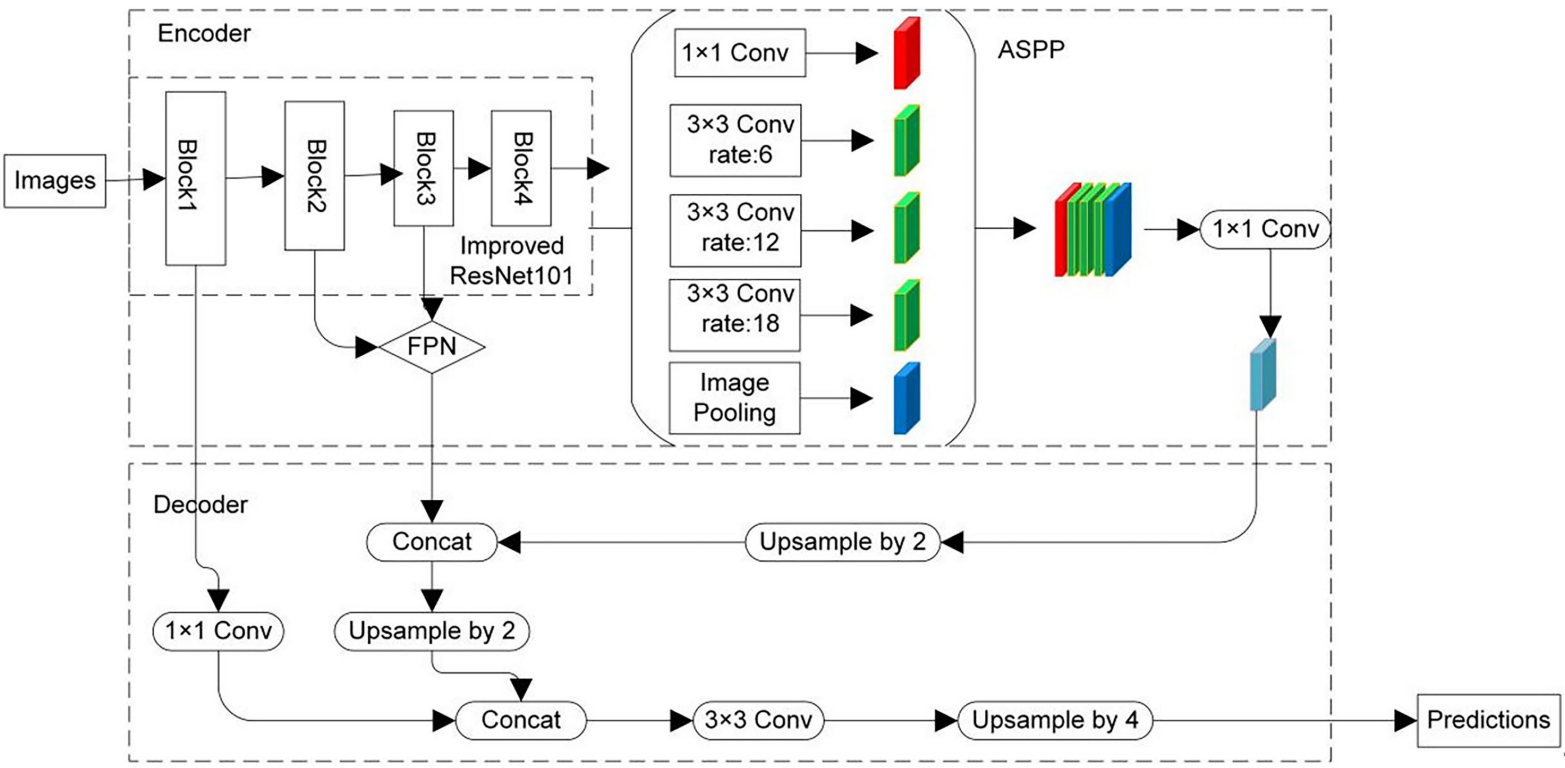
Improved DeepLab v3+ network structure.

**Figure 5:**
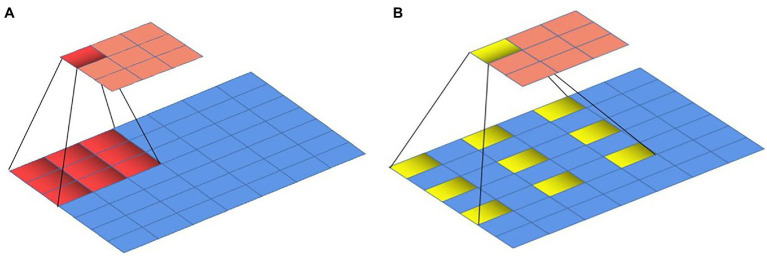
Convolution execution process. **(A)** Standard convolution work process, **(B)** The dilated convolution work process.

The improved DeepLab v3+ network consists of two parts, an encoder and decoder (Chen et al., 2018), which shows in Figure 4. The encoder part trains the network, progressively obtains the feature maps, and captures higher-level semantic information. The decoder part semantically projects the features learned by the encoder part into the pixel space to achieve pixel segmentation. In the encoder, the backbone network is constructed using ResNet101 and the ECA module is inserted in its residual module. Moreover, to enhance the semantic information of the feature map, the feature maps of Block2 and Block3 of the ResNet101 network are fused. Atrous Spatial Pyramid Pooling (ASPP; Chen et al., 2018) is connected behind the ResNet101 backbone network. Dilated convolution with different sampling rates can be sampled in parallel by ASPP, which is equivalent to capturing the context of images at multiple scales. Dilated convolution (Yu et al., 2017) adds atrous to the convolution map during the convolution operation to expand the reception field so that each convolution output can contain a larger range of information. In addition to the convolution kernel, the dilated convolution also has a hyper-parameter dilation rate. It refers to the number of intervals between the convolution kernel during convolution mapping, that is, the number of atrous inserted. Figure 5 shows the execution process of convolution. Here, Figure 5A is the standard convolution process and Figure 5B is the process of dilated convolution.

The encoder module has three outputs. The first is the low-level feature (LF) output by Block1 in the backbone network. The second is the fusion feature (FF) of Block2 and Block3 output by the FPN. The last one is the high-level feature (HF) output by the ASPP module after 1 × 1 convolution. High-level feature output concatenates to FF after it has undergone 2-fold up-sampling, and then the second 2-fold up-sampling is performed. The result of this operation is concatenated to the LF, which has been convoluted by 1 × 1 convolution. A 3 × 3 convolution is performed after the above operation, and then a single four-fold up-sampling is performed. Then, the dense classification of pixels is obtained, which is image segmentation.

### Parameters Setting of Improved DeepLab v3+ Network

The stochastic gradient descent method was applied to the end-to-end training of the deep learning network, and the loss function was set to Dice_Loss as shown in Equation (1). The weight decay rate was set to 0.001, and the kinetic energy factor was set to 0.8. The initial learning rate was set to 0.001, the learning rate decay mode was exponential decay, and the Batch_size was set to 4. The maximum iteration period (Epochs) was set to 120, and the network input size was set to 512 × 512. The data set was stored in the format of the VOC 2007 data set, and pre-trained model weights were loaded in the experiment to speed up the convergence of the model.


(1) $$Dice\_Loss=\frac{FP+FN}{FP+2TP+FN}$$

where TP represents the true positives, indicating that the black rot area of grape leaves automatically segmented by the model overlaps with the real disease area; FP represents the false positives, indicating that the model misidentified the background area as a black rot spot area and segmented it; TN represents the true negatives, indicating that the model identified the real background area as the background area; and FN represents the false negatives, indicating that the model misidentified the real black rot area as the background area.

### Evaluation Indicators

In this study, to evaluate the performance of the improved DeepLab v3+ network segmentation, the mean intersection over union (mIOU), the dice coefficient (Dice), the pixel accuracy (ACC), precision (*P*), recall (*R*), and *F1*-score were selected as evaluation metrics.

The mIOU is a common evaluation metric in semantic segmentation methods. In semantic segmentation, the predicted and true regions are obtained by pixel operation, and Equation (2) is as follows:


(2) $$\mathrm{mIOU}=\frac{1}{2}\overset{1}{\underset{i=0}{\sum }}\frac{p_{\mathrm{ii}}}{\overset{1}{\underset{j=0}{\sum }}p_{ij}+\overset{1}{\underset{j=0}{\sum }}p_{ji}-p_{ii}}$$

where p*_ij_* denotes the number of pixels that originally belonged to class *i* but are predicted to be class *j*, p*_ii_* denotes the number of pixels whose true label is class *i* predicted to be class *i*, and p*_ji_* denotes the number of pixels that originally belonged to class *j* but are predicted to be class *i*. In this study, the pixels in each image were classified into two classes: black rot spots and background.

The Dice value is usually used to calculate the similarity of two samples, and the value range is (0,1). A Dice value close to 1 indicates a high set similarity, that is, the target is better segmented from the background; while a Dice value close to 0 indicates that the target cannot be effectively segmented from the background. The dice value equation is as follows:


(3) $$Dice=\frac{2TP}{FP+2TP+FN}$$

The ACC is the ratio of the number of correctly predicted pixels to the total number of pixels in the category, and its equation is as follows:


(4) $$ACC=\frac{TP+TN}{TP+FN+FP+TN}$$

The *P*, *R*, and *F1*-score were calculated by the following equation:


(5) $$\{\begin{array}{c} P=\frac{TP}{TP+FP}\\ R=\frac{TP}{TP+FN}\\ F1-score=2\times \frac{P\cdot R}{P+R} \end{array}$$

### Comparison of the Effects of Different Improvements of DeepLab v3+

To verify the effectiveness of the neural network constructed in this paper for grape leaf spot segmentation, eight sets of comparison experiments with different improvements were designed. These eight different improvements were named from Imp1 to Imp8, as shown in Table 1. In Imp1, the three dilated convolutions of the ASPP model of the original DeepLab v3+ network were modified to four dilated convolutions, and their dilated rate sizes were 4, 8, 12, and 16, respectively. Theoretically, the increase of dilated convolutions and the change of dilated rate sizes will improve the fusion effect of semantic features. In Imp2, the ResNet 101, backbone of the DeepLab v3+, was replaced with Wide ResNet (Zagoruyko and Komodakis, 2016), which can improve the network segmentation performance by changing the width of the network without changing the network depth. The residual module of the backbone ResNet101 was inserted into the ECA module in Imp3, and the ECA model can adaptively adjust the convolutional kernel size in each channel of the residual block, which can improve the segmentation effect of the network. The coding side of the DeepLab v3+ network was added with a feature fusion branch based on the FPN in Imp4. The FPN can fuse different levels of feature maps and can obtain feature maps that can reflect semantic information at different scales. In imp5, the ASPP part of DeepLab v3+ was combined with DenseNet (Yang et al., 2018) to form DenseASPP, and the new module had a larger receiver field and more densely sampled points. Imp1, Imp3, and Imp4 were combined as Imp6. Imp3 and Imp5 were combined as Imp7. Imp3 and Imp4 were combined as Imp8, which is the improvement method used in this paper.

**Table 1:** Different DeepLab v3+ improvement methods.

Improvement methods	Improvement content
Imp1	Modify the three dilated convolutions of ASPP in the original network to four dilated convolutions with a dilated rate size of 4, 8, 12, and 16, respectively
Imp2	Replace the ResNet backbone in the original network with wider ResNet
Imp3	Insert the ECA module in the residual module of the backbone ResNet101
Imp4	A feature fusion branch based on an FPN is added to the coding side of the original network
Imp5	Combine the ASPP part of the original network with DenseNet to form DenseASPP
Imp6	Imp1 + Imp3 + Imp4
Imp7	Imp3 + Imp5
Imp8	Imp3 + Imp4

## Results

### The Segmentation Results of Improved DeepLab v3+ for Grape Leaves Black Rot

**Figure 6:**
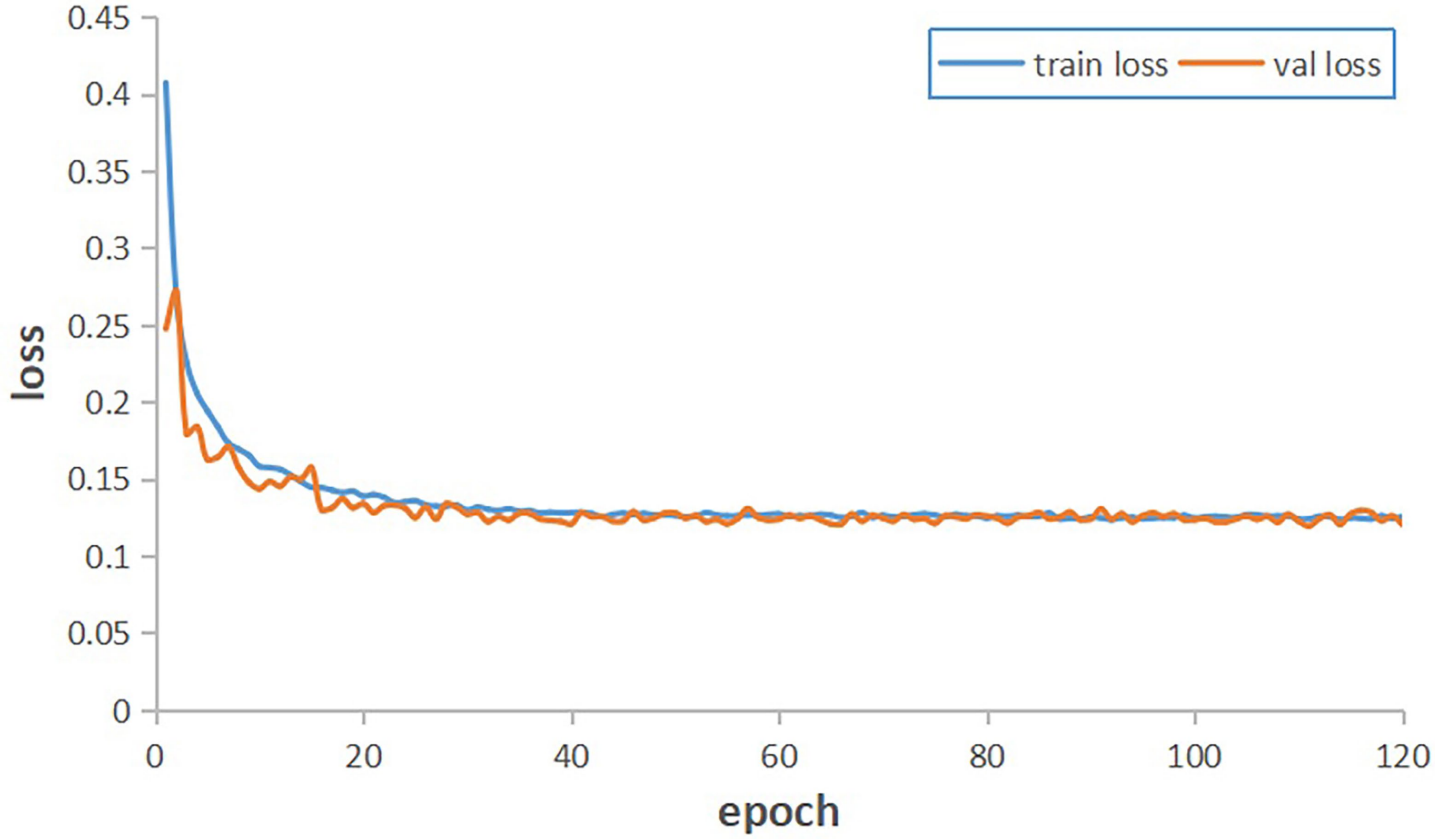
Improved DeepLab v3+ training results.

The training dataset with annotation information was fed into the improved DeepLab v3+ network for training. The network was trained for 120 epochs, which required around 8.3 h. During the training process, the training model was saved once every 1 epoch, and a total of 120 completed models were saved. The convergence of the model can be reflected by the loss values generated during the training process. Figure 6 shows the changes in the loss values of the training data and validation data in the training set during the training process. The training loss and validation loss gradually converged to stability during the training process, and the final training loss and validation loss values stabilized at 0.132.

**Figure 7:**
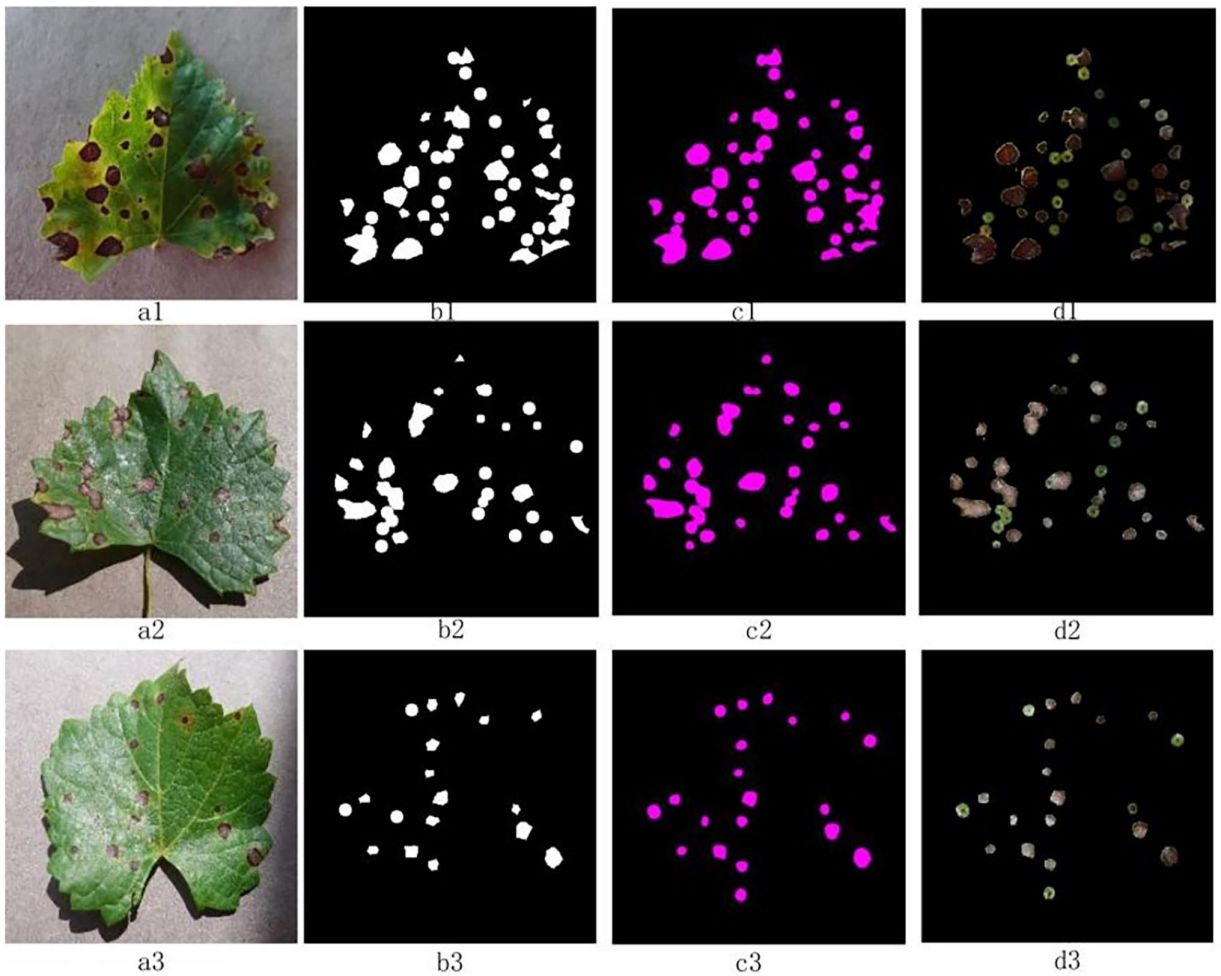
Segmentation effects of the improved DeepLab v3+ on the test set TS1 image. The “a” column is the original image, the “b” column is the labeled mask, the “c” column is the segmentation result of the model, and the “d” column is the disease spot extraction result.

To verify the performance of the model, the optimal model at the end of training was selected to be used for segmentation trials on test set TS1. The statistical results of the experiment before and after improved DeepLab v3+ are shown in Table 2. As can be seen from Table 2, the improved DeepLab v3+ outperforms the pre-improvement DeepLab v3+ in all evaluation metrics. In particular, it improved 3.0, 2.3, and 1.7% in mIOU, *R*, and *F1*-score, respectively. The effects of the segmentation are shown in Figure 7.

**Table 2:** Statistics of the segmentation results of the test set TS1 by the before and after improved DeepLab v3+.

Algorithm	Evaluation indicators					
	mIOU	ACC	Dice	*P*	*R*	*F1*-score
DeepLab v3+	0.823	0.984	0.903	0.949	0.861	0.903
DeepLab v3+ (improved)	0.848	0.987	0.918	0.957	0.881	0.918

**Figure 8:**
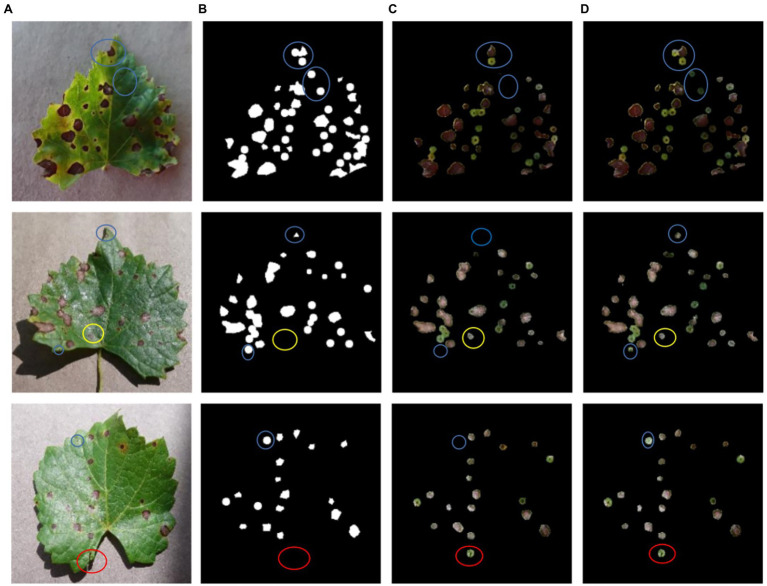
A comparison of network training results before and after DeepLab v3+ improvement. **(A)** The original image, **(B)** the manually labeled and segmented image, **(C)** the DeepLab v3+ segmentation results, **(D)** the improved DeepLab v3+ segmentation results.

Figure 8 shows the segmentation results of DeepLab v3+ before and after improvement applied to black rot spots of grape leaves in test set TS1. Figure 8A shows the original image, Figure 8B shows the manually labeled and segmented image, Figure 8C shows the segmentation results of DeepLab v3+ before improvement, and Figure 8D shows the segmentation results of DeepLab v3+ after improvement. The blue markers in Figure 8 indicate the small spots targeted in the original image that were not identified and segmented by the original network model but were correctly segmented by the improved network model. The yellow markers indicate that the semantic segmentation network correctly identified and segmented some small spots in the original image even though they were not manually labeled and segmented due to human oversight. This also demonstrates that the use of deep learning methods can reduce subjective errors caused by manual segmentation. The red markers indicate that the leaf edges were misidentified as spots and segmented by the network model due to shadows. This indicates that there is a requirement for background conditions for disease spot recognition using deep learning. Furthermore, Figure 8 shows that although the improved network model could segment the spots at the same location, the improved network model was more accurate and the segmented spots overlapped more with the actual spots.

**Figure 9:**
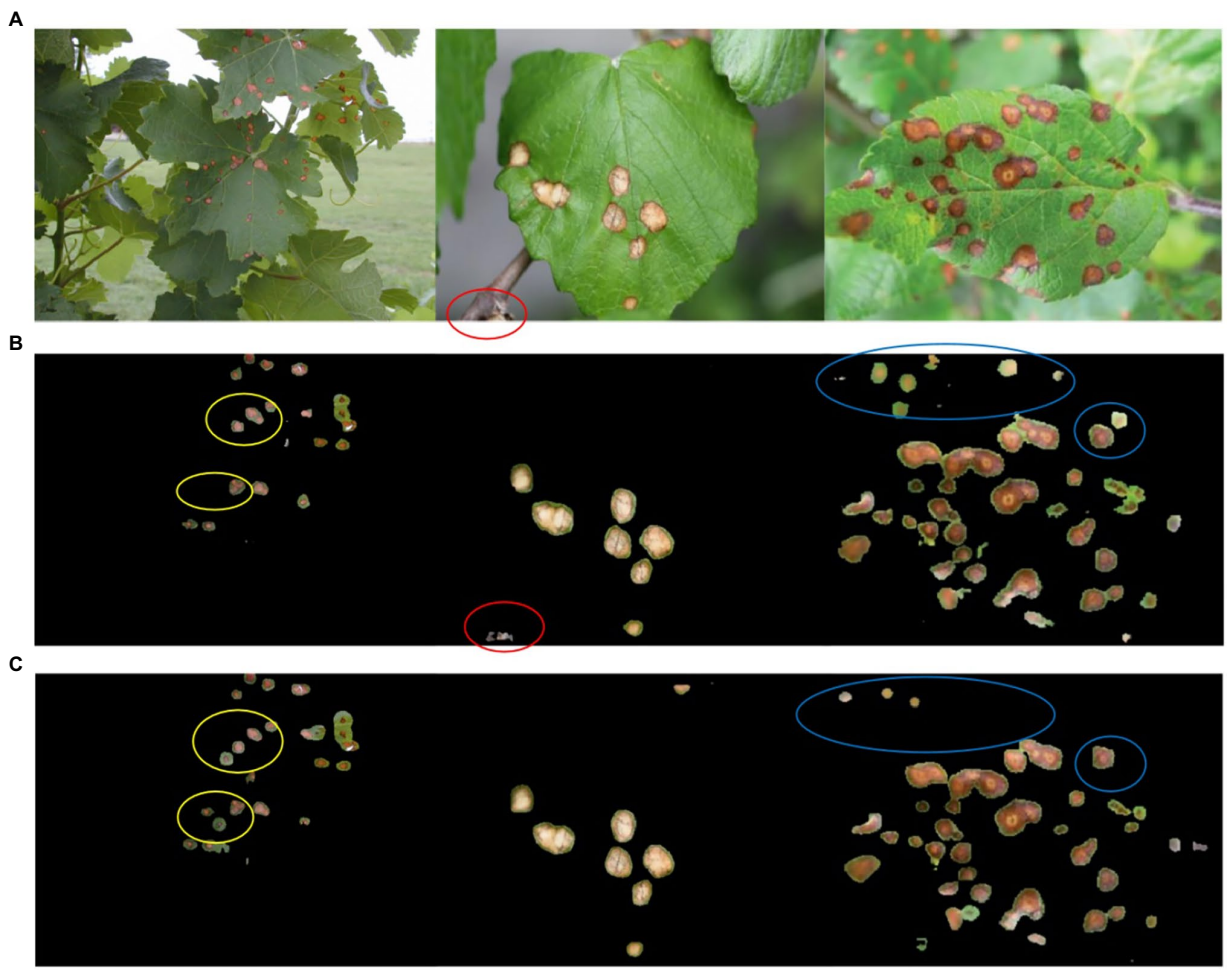
A comparison of segmentation results of test set TS2 images before and after improvement of DeepLab v3+. **(A)** The original figure, **(B)** the segmentation results of DeepLab v3+ without improvement, **(C)** the segmentation results of the improved DeepLab v3+.

Experiments with the Plant Village dataset demonstrated that the improved DeepLab v3+, which incorporates an attention mechanisms and feature pyramids, could improve the segmentation of black rot spots on grape leaves. An additional dataset, TS2, with 108 images from photos taken in different orchard fields was used for testing to verify the effectiveness of the method in an orchard field setting. The TS2 dataset was tested experimentally using the DeepLab v3+ network before and after the improvement. Figure 9 shows the experimental results of the DeepLab v3+ algorithm before and after the improvement on TS2. Figure 9A is the original image, Figure 9B is the unimproved DeepLab v3+ segmentation result, and Figure 9C is the improved DeepLab v3+ segmentation result. To show the network segmentation effect before and after the improvement, different colors are marked in Figures 9B,C. The yellow markers show that the improved network was more comprehensive in terms of the segmentation effect. The red markers show that the improved network was more accurate in segmentation. The blue markers show that the improved network was less affected by the background under the interference of complex background. The experimental results show that the improved DeepLab v3+ network performed better than the unimproved DeepLab v3+ network. Moreover, comparing the experimental segmentation effects shows that the improved DeepLab v3+ network can be applied to an actual orchard situation.

The statistical results of DeepLab v3+ before and after the improvement are shown in Table 3 for test set TS2. Table 3 shows that the improved DeepLab v3+ did not segment as well as TS1 for grape leaf black rot spots in a natural environment. This is because the images in TS1 were indoor environments, and the grape leaves were tiled with a single and simple background. In contrast, there were negative effects, such as overlapping leaves, gaps formed by shading, and lighting in the orchard field environment, which caused interference for accurate segmentation. Moreover, for large and dense spot areas, the network model would segment the dense spot areas as a whole; thus incorrectly classifying some backgrounds as spot areas. However, segmentation using the improved DeepLab v3+ still outperformed the one before the improvement, especially reaching scores of 0.756, 0.734, and 0.805 in mIOU, *R*, and *F1*-score, respectively, which were 3.3, 2.5, and 1.9% higher than those before improvement. This indicates that the proposed method improves the segmentation performance of DeepLab v3+, and its ubiquity and adaptability for application in a real environment are better compared with the unimproved network model.

**Table 3:** Statistics of the segmentation results of test set TS2 images before and after DeepLab v3+ improvement.

Algorithm	Evaluation indicators					
	mIOU	ACC	Dice	*P*	*R*	*F1*-score
DeepLab v3+	0.732	0.874	0.845	0.916	0.785	0.845
DeepLab v3+ (improved)	0.756	0.889	0.861	0.925	0.805	0.861

### Comparison of the Effects of Different Improvements of DeepLab v3+

For the above eight DeepLab v3+ improvement methods, the same training set was used for training, and the performances were tested with the test set TS1. To compare the results of different improvement methods, the parameters of the network, such as the learning rate, epoch, and batch size, were kept consistent during the experiments. The test results are shown in Table 4, where the four parameters mIOU, ACC, Dice, *P*, *R*, *F1*-score, and Pt are used for comparison. The Pt is the storage space occupied by the weight file generated after network training. Table 4 shows that the performance indicators of the unimproved DeepLab v3+ on the test set TS1 were 0.823, 0.984, and 0.811 for mIOU, ACC, and Dice, respectively. Table 5 shows that, compared with the DeepLab v3+ network before improvement, the scores of mIOU, ACC, and Dice were higher for the other six of the eight improved methods, except for Imp1 and Imp2. Compared with the DeepLab v3+ before improvement, Imp3 and Imp4 were 1.6% and 1.3% higher in mIOU and 0.5% and 1.3% higher in Dice, respectively. This indicates that fusing ECA or adding FPN in DeepLab v3+ network could improve the segmentation performance of the model. Although the improved method of Imp5 had improved mIOU and Dice by 1.4% and 1%, respectively. The Pt generated by this method required more memory space than that of Imp3 and Imp4. Moreover, Imp6 is a fusion of Imp1, Imp2, and Imp3, but its mIOU and Dice were lower than Imp3 and Imp4. This shows that the additional change of the dilated rate of the dilated convolution did not improve the performance of the network, which was consistent with the test results of Imp1. Besides, Imp7 is a fusion of Imp3 and Imp5, because fusing ECA in Imp3 alone or modifying ASPP to DenseASPP in Imp5 alone could improve network performance. Thus, Imp7 scored higher in mIOU than Imp3 and Imp5, and the Dice value was in line with Imp5 and higher than Imp3. However, the introduction of DenseASPP led to a larger computation within the network and its obtained weight file was relatively large, which was consistent with the performance of Imp5. The final improved method adopted in this paper was Imp8, which fuses Imp3 and Imp4 and adds both ECA and FPN in the DeepLab v3+ network. Here, Imp8 scored 0.848, 0.987, 0.918, 0.957, 0.881, and 0.918 for mIOU, ACC, Dice, P, *R*, and *F1*-score, respectively, after the same test set test, and it received the highest scores among all eight methods. Moreover, its weight file occupied 241,553 kb of space, which was in the middle level among the eight improved methods. This indicates that the Imp8 method used in this paper has a better overall performance compared to other improvement methods.

**Table 4:** Comparison of the test results of different improvement methods of DeepLab v3+.

Type	Evaluation indicators						
	mIOU	ACC	Dice	*P*	*R*	*F1*-score	Pt (kb)
Imp1	0.812	0.982	0.896	0.945	0.852	0.896	572,794
Imp2	0.818	0.982	0.900	0.947	0.857	0.900	554,101
Imp3	0.839	0.985	0.912	0.954	0.874	0.912	232,841
Imp4	0.836	0.987	0.911	0.953	0.872	0.911	241,541
Imp5	0.837	0.986	0.911	0.954	0.873	0.911	310,161
Imp6	0.833	0.986	0.909	0.952	0.869	0.909	241,533
Imp7	0.841	0.986	0.914	0.955	0.876	0.914	310,173
Imp8	0.848	0.987	0.918	0.957	0.881	0.918	241,553

**Table 5:** Detection statistics results of the two methods for the grape leaves in Figure 11.

Leaf	Number of real disease spots			Pixels of real disease spots			Actual number	Detected by the detection method	Detected by the segmentation method	Actual pixels	Segmented by the detection method	Segmented by the segmentation method
Left	18	16	18	2,301	/	2,237
Middle	17	10	16	2,328	/	2,228
Right	14	12	12	2,132	/	2,066

*The disease spots were manually segmented using LabelMe and then the number of pixels was counted by a self-developed python program. All these operations were carried out under the guidance and supervision of the grape disease specialist*.

**Figure 10:**
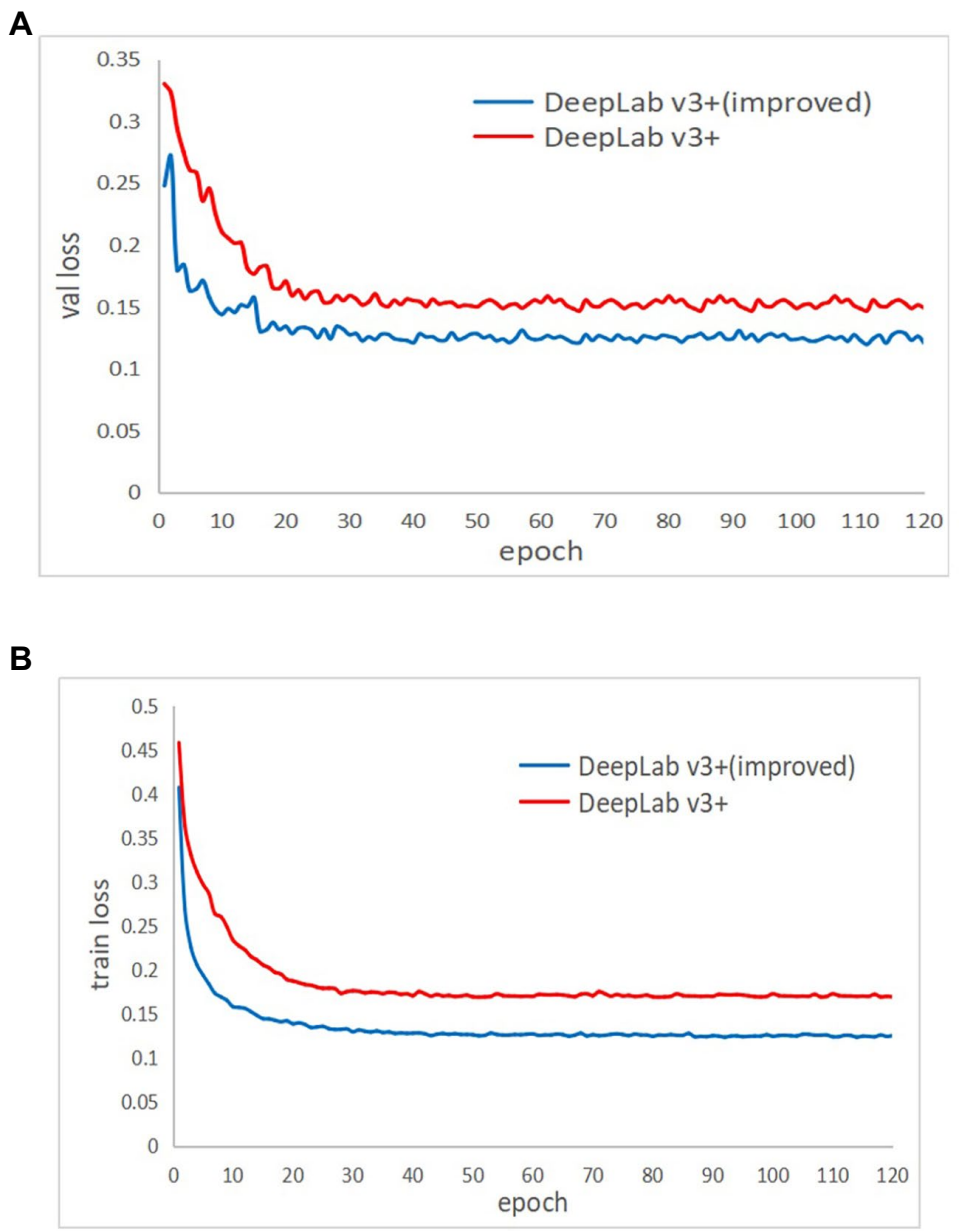
Comparison of the training results of the network before and after the improvement of DeepLab v3+. **(A)** The training set, **(B)** the validation set.

A comparison of the training performance of the unimproved DeepLab v3+ and the improved network using the Imp8 method is shown in Figure 10. The training set loss curves are shown in Figure 10A, where the red curve is before improvement and the blue curve is after improvement. When training until the model converged, the value of the red curve was about 0.17 and the value of the blue curve is about 0.132, which indicates that the improved model fit better on the training set than before improvement. Figure 10B shows the validation set loss curves, where the red curve is before improvement and the blue curve is after improvement. When training until the model converged, the value of the red curve was about 0.16, while the value of the blue curve was about 0.13, which indicates that the generalization ability of the model after the improvement was better than that before the improvement. Therefore, the improved DeepLab v3+ always converged faster and had better model fitting ability than the pre-improvement one whether on the training set or the validation set.

## Discussion

### Effect Comparison Between Detection and Segmentation for Disease Spots

**Figure 11:**
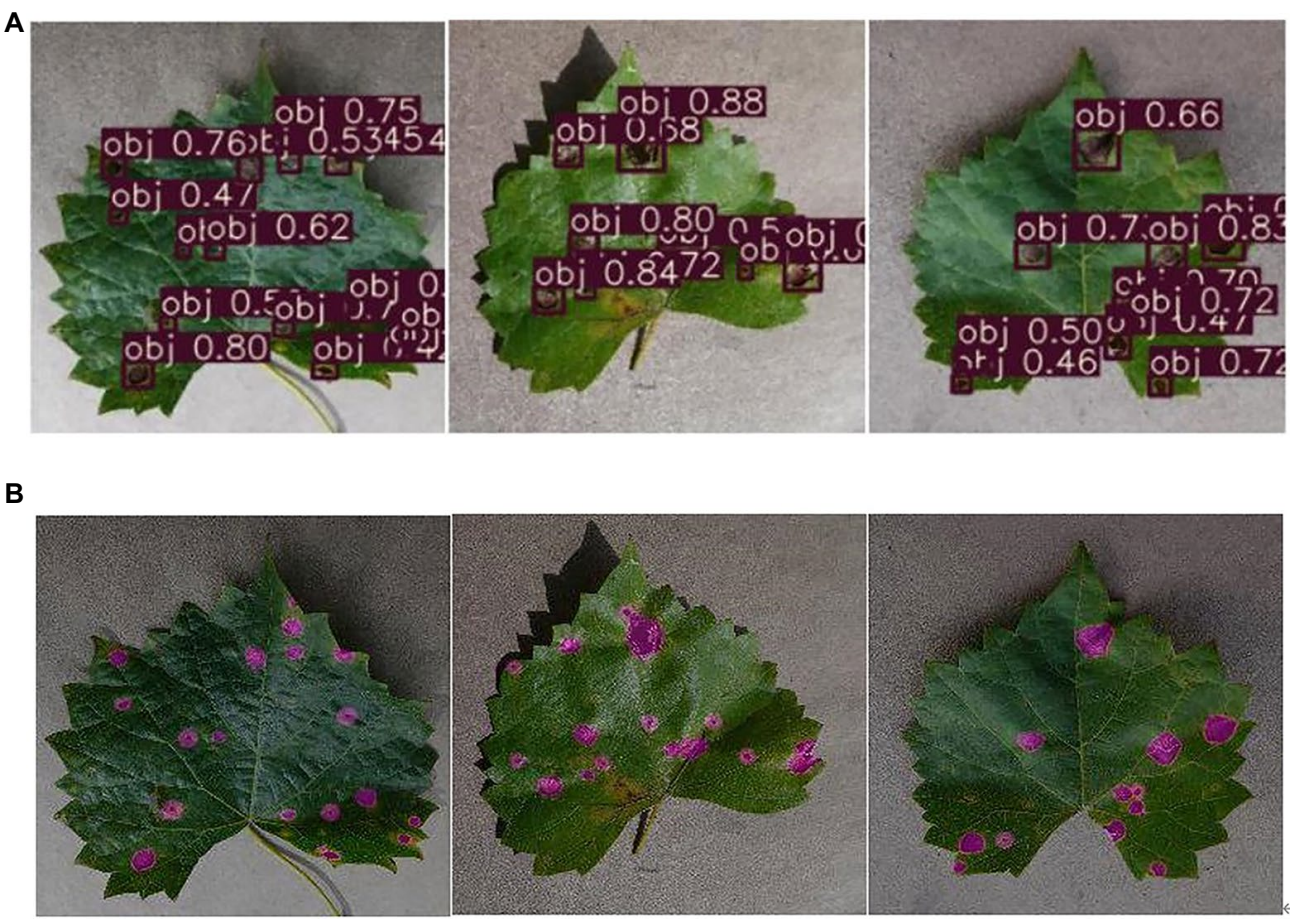
The effect comparison between detection and segmentation on diseased spot. **(A)** The results of disease spots detection, **(B)** the results of disease spots segmentation.

The grape leaf black rot disease spots can be recognized in the previous research of our group, and the spots were accurately segmented from the background in this paper. The effect of disease spots detection and segmentation for test set TS1 is compared in Figure 11. Figure 11A shows the result of detection using the previous recognition method (Zhu et al., 2021), the number and location of the disease spots can be recognized, but cannot be segmented from the background. Figure 11B shows the result of segmentation using the method in this paper. The disease spots are not only recognized but also segment from the background according to their contour shape. Table 5 shows the detection statistics results of the two methods for the grape leaves in Figure 11. As shown in Table 5, the segmentation method not only recognizes the number of disease spots but also obtains the number of pixels of spots. In addition, the segmentation method also detects and segments some tiny spots, which shows that this method is also better than the previous methods in recognition performance.

### Comparison of Different Segmentation Algorithms

In this paper, DeepLab v3+ was chosen as the base algorithm to be improved for the segmentation of grape leaf black rot spots. This choice was based on the comparison of three common current mainstream deep learning segmentation algorithms. Pyramid Scene Parsing Network (PSP Net; Zhao et al., 2017) and U-Net are the other two common deep learning segmentation methods besides DeepLab v3+. PSPNet consists of a ResNet backbone that imposes a dilated convolution and a pyramid pooling module, which can mine global contextual information for fast network training. U-Net is an FCN with a simple structure, which can obtain very accurate segmentation results using few training images and is widely used in medical image analysis.

**Figure 12:**
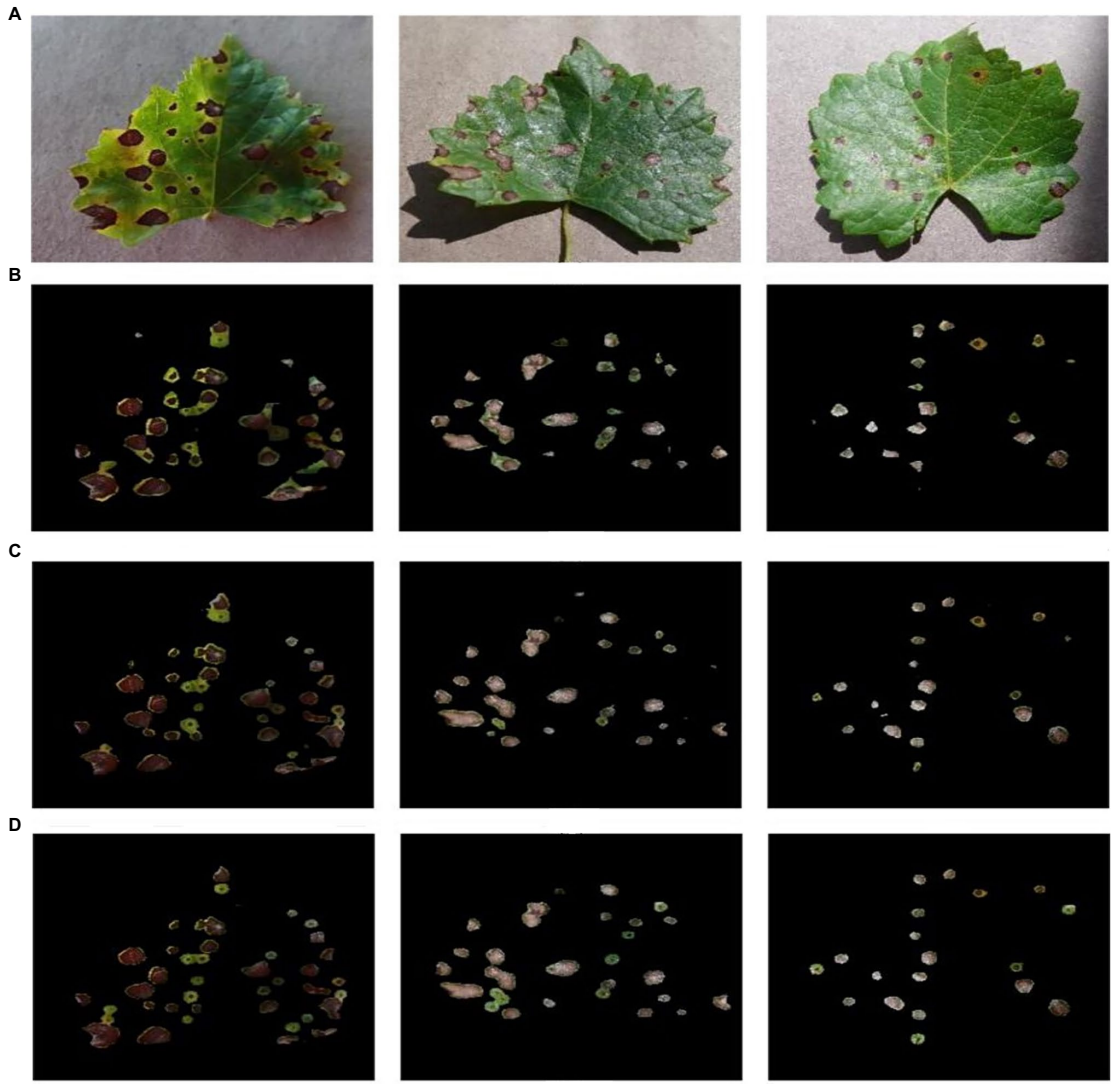
Comparison of the segmentation results of different segmentation algorithms on the test set TS1 images. **(A)** The original image, **(B)** the PSP Net segmentation and extraction results, **(C)** the U-Net segmentation and extraction results, **(D)** improved DeepLab v3+ segmentation and extraction results.

In this study, these three semantic segmentation networks were trained using the same dataset, and segmentation experiments of black rot spots were conducted on the test set TS1. Figure 12 shows the segmentation results of three different networks. As shown, PSPNet could segment the black rot spots, but the network performed poorly for the segmentation of connected spots, and it mistakenly segmented the leaf part between two spots. The segmentation effect of U-net was better than PSPNet, which could separate the lesion area independently, but the segmentation was not fine enough. Improved DeepLab v3+ was better than the other two methods.

Table 6 shows the experimental statistical results of the different segmentation methods. In terms of ACC, there was no significant difference between the three methods, but in the mIOU metric, improved DeepLab v3+ was 10.6 and 4.4% higher than PSPNet and U-net, respectively. In terms of the *R* value, improved DeepLab v3+ was 8.2 and 3.4% higher than PSPNet and U-net, respectively. The experimental results showed that the improved DeepLab v3+ had better segmentation performance compared with PSPNet and U-net, and the improved DeepLab v3+ could further improve the segmentation performance of black rot spots on grape leaves.

**Table 6:** Statistical segmentation results of different segmentation algorithms on the test set TS1 images.

Algorithm	Evaluation indicators					
	mIOU	ACC	Dice	*P*	*R*	*F1*-score
PSP Net	0.767	0.972	0.868	0.929	0.814	0.868
U-Net	0.812	0.98	0.896	0.945	0.852	0.896
DeepLab v3+ (improved)	0.848	0.987	0.918	0.957	0.881	0.918

## Conclusion

This paper proposes an improved DeepLab v3+ network model for the segmentation of black rot spots on grape leaves. This method inserts the ECA module into the residual module of the original DeepLab v3+ backbone network. Moreover, a feature fusion branch based on a FPN is added at the encoder end. One 4-fold up-sampling to two 2-fold up-sampling are modified in the original network. To verify the performance of the improved network model, two test sets based on Plant Village and an orchard field environment were constructed for experiments. The experimental results showed that the improved DeepLab v3+ network model exhibited better performance on both test sets than before improvement, and the improved model could be applied to the segmentation of black rot spots on grapes in real production environments. This approach can not only provide an effective tool for classifying grape disease extent classes but also be applied to the evaluation of other plant leaf and fruit diseases. In future work, we will attempt to combine super-resolution image enhancement with this approach to further improve the effect of small target recognition and segmentation.

## Data Availability Statement

The raw data supporting the conclusions of this article will be made available by the authors, without undue reservation.

## Author Contributions

HY, JZ, and MC conceived the idea and proposed the method. JZ and QW contributed to the preparation of equipment and acquisition of data. JZ wrote the code and tested the method. JZ, QW, and MC validated results. HY and JZ wrote the paper. HY, JZ, and ZC revised the paper. All authors have read and approved the final manuscript.

## Funding

This work was supported by the National Natural Science Foundation of China (No. 32001412), the Key Research and Development Program of Hebei Province (19227206D).

## Conflict of Interest

The authors declare that the research was conducted in the absence of any commercial or financial relationships that could be construed as a potential conflict of interest.

## Publisher’s Note

All claims expressed in this article are solely those of the authors and do not necessarily represent those of their affiliated organizations, or those of the publisher, the editors and the reviewers. Any product that may be evaluated in this article, or claim that may be made by its manufacturer, is not guaranteed or endorsed by the publisher.
